# A study on the differential of solid lung adenocarcinoma and tuberculous granuloma nodules in CT images by Radiomics machine learning

**DOI:** 10.1038/s41598-023-32979-6

**Published:** 2023-04-11

**Authors:** Huibin Tan, Ye Wang, Yuanliang Jiang, Hanhan Li, Tao You, Tingting Fu, Jiaheng Peng, Yuxi Tan, Ran Lu, Biwen Peng, Wencai Huang, Fei Xiong

**Affiliations:** 1 Department of Radiology, The General Hospital of Central Theater Command, PLA, Wuhan, 430070 Hubei China; 2 School of Computer Science and Information Engineering, Hubei University, Wuhan, 430062 Hubei China; 3 Medical School, Hubei Minzu University, Enshi, 445000 Hubei China; 4 School of Basic Medical Sciences, Wuhan University, Wuhan, 430060 Hubei China

**Keywords:** Cancer, Cancer imaging, Lung cancer

## Abstract

To study the classification efficiency of using texture feature machine learning method in distinguishing solid lung adenocarcinoma (SADC) and tuberculous granulomatous nodules (TGN) that appear as solid nodules (SN) in non-enhanced CT images. 200 patients with SADC and TGN who underwent thoracic non-enhanced CT examination from January 2012 to October 2019 were included in the study, 490 texture eigenvalues of 6 categories were extracted from the lesions in the non-enhanced CT images of these patients for machine learning, the classification prediction model is established by using relatively the best classifier selected according to the fitting degree of learning curve in the process of machine learning, and the effectiveness of the model was tested and verified. The logistic regression model of clinical data (including demographic data and CT parameters and CT signs of solitary nodules) was used for comparison. The prediction model of clinical data was established by logistic regression, and the classifier was established by machine learning of radiologic texture features. The area under the curve was 0.82 and 0.65 for the prediction model based on clinical CT and only CT parameters and CT signs, and 0.870 based on Radiomics characteristics. The machine learning prediction model developed by us can improve the differentiation efficiency of SADC and TGN with SN, and provide appropriate support for treatment decisions.

## Introduction

Since 1990s, the change of tumor spectrum caused by environmental deterioration and habits has attracted increasing attention of the international community^1,2^. The incidence rate of lung invasive adenocarcinoma is increasing dramatically in recent years, especially in China^3^. At present, the lung tumor ranks first in the malignant tumor spectrum in China. Lung invasive adenocarcinoma is a serious public health problem because of its high incidence rate and the lack of specificity of the population. It is estimated that there are more than 2000 cases of lung invasive adenocarcinoma in China every year. Studies have shown that the survival time of patients with invasive adenocarcinoma who are treated early, quickly, and effectively will be prolonged, and correct diagnosis can also avoid overtreatment of patients with non-lung invasive adenocarcinoma^4^. Therefore, early, and accurate diagnosis of lung invasive adenocarcinoma is vital for patients with atypical thoracic CT manifestations of SN.

In the past, the research on lung invasive adenocarcinoma mostly focused on adenocarcinoma in situ (AIS) and minimally invasive adenocarcinoma^5^ with ground glass nodules (GGN), and a lot of achievements have been made^5–11^. Pulmonary infiltrating adenocarcinoma with SN is often asymptomatic. It is occasionally found by routine thoracic CT examination because of chest pain and cough. However, most lung infiltrating adenocarcinoma is found by chance in physical examination or other examinations (such as abdominal CT, coronary CTA, or aortic CTA). When the CT features of solitary pulmonary nodules cannot clearly distinguish lung infiltrating adenocarcinoma from other diseases with solitary pulmonary nodules, clinicians frequently treat them according to the consensus of experts on the treatment of lung infiltrating adenocarcinoma4. Among them, the most common overtreatment is to treat tuberculous granuloma according to the scheme of lung infiltrating adenocarcinoma. In the conventional thoracic CT images, pulmonary infiltrating adenocarcinoma and tuberculous granuloma are overlapped in the following aspects: lobulation sign, pleural adhesion sign, spiculation sign, bronchiole perforation deformation, vacuole sign, small vessel perforation sign, hilar lymphadenopathy^12,13^. There is a great overlap between the two diseases in CT images, so it is difficult to distinguish them with naked eyes.

Radiomics proposed by Lambin in 2012 is to improve the ability of the decision support model by combining the extracted CT image features with other available patient data, which is an extension of computer-aided diagnosis^14,15^. Numerous studies have shown that Radiomics has been successfully applied to the differential diagnosis, staging and evaluation of lung, liver, and kidney lesions, especially in the differential diagnosis of tumors. Therefore, it is possible to differentiate lung infiltrating adenocarcinoma and tuberculous granuloma with solitary pulmonary nodules by Radiomics^16,17^.

Thus, our aim is to establish a Radiomics classifier based on the texture features of CT images to differentially diagnose lung infiltrating adenocarcinoma and tuberculous granulomas that appear as solitary pulmonary nodules in CT images.

## Materials and methods

### Ethical recognition and informed consent

This study is based on the principles of the Helsinki Declaration. The informed consent of all subjects and/or their legal guardians has been obtained for this study. The Ethics Committee of the General Hospital of the Central Theater of the People's Liberation Army approved the study. (Scheme/Decision No. [2020] 035-1).

### Patient

We collected patients with invasive adenocarcinoma of the lung and patients with tuberculous granuloma who were diagnosed pathologically in our institution from January 2012 to May 2019. These patients had records of demographic data including age, gender, smoking status, and smoking index^18^. The inclusion and exclusion criteria and the study flow for patient enrollment are listed in the data supplement.

Finally, 200 patients were included in the study, of which 112 were diagnosed as lung invasive adenocarcinoma. In the process of machine learning, the classifier randomly divided all 200 patients into two queues according to the ratio of 8:2, including 140 patients in the primary data set and 60 patients in the validation data set.

### Doctor

We invited two radiologists with 10 years of diagnostic experience who are not our research team to study and segment CT images.

### CT image acquisition

Thoracic CT images were obtained with a 320-row CT scanner (320 CT, Aquilion ONE; Toshiba Medical Systems, Tokyo, Japan). Scanning range: upper edge of 6th cervical vertebra to 2nd lumbar vertebra. Scanning parameters: rotating speed of spherical tube 0.625 s/rot, pitch 1, tube voltage 100–120 kv, adaptive tube current technology (110–140mas). Reconstruction parameters: 0.5 mm slice thickness and 0.5 mm interval, window level − 550HU, window width 1500HU and average density projection mode.

Image processing and texture extraction. We use free and open-source imaging biomarker explorer (IBEX) developed at MD Anderson Cancer Center (Houston, TX, USA) for CT image segmentation and texture extraction. The thoracic CT images of 200 patients were randomly assigned to 2 radiologists for manual segmentation, to confirm the volume of interest (VOI) of the nodules.

**Figure 1:**
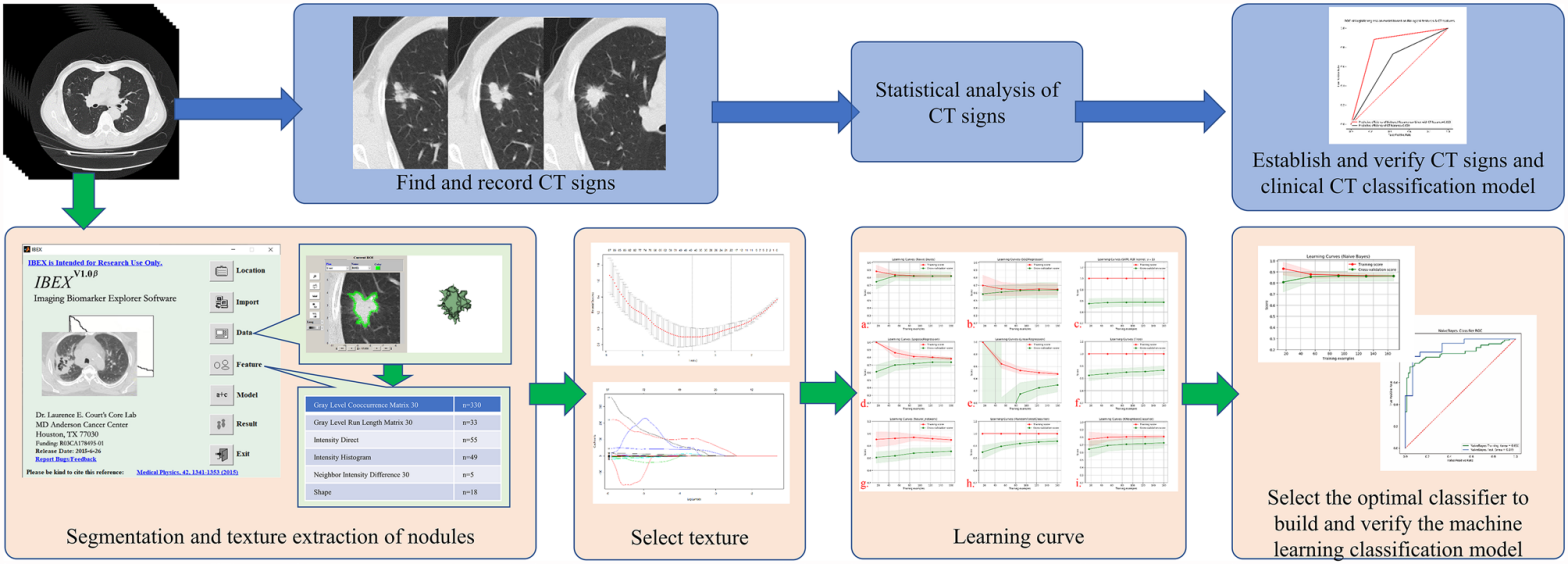
Research path map. Blue path, routine clinical CT diagnosis process. The features were found from CT images, and the differential diagnosis models were established to evaluate CT features and clinical CT diagnosis. Green path, machine learning process. The texture features are extracted from CT images by IBEX software. The extracted texture features are reduced by LASSO regression. The best classifier is selected by learning curves of machine learning, and the machine learning classification model is established and tested.

**Figure 2:**
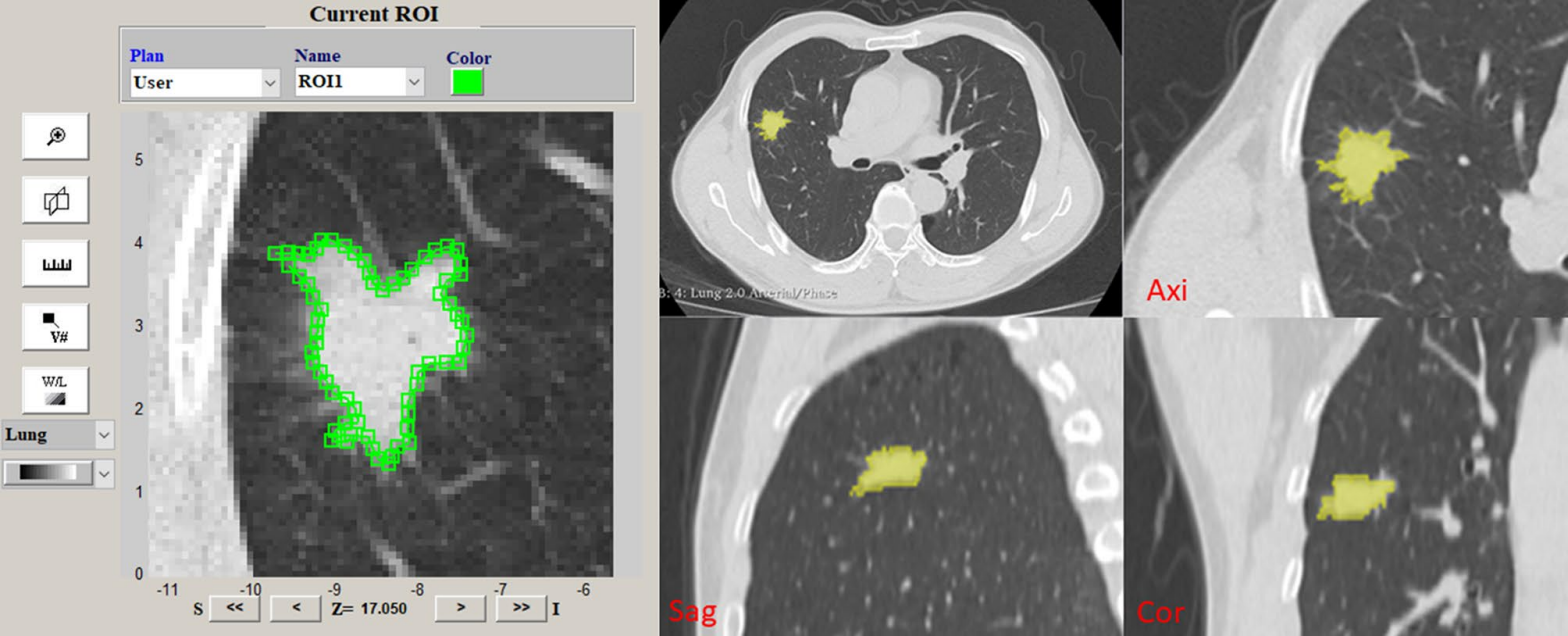
Image segmentation diagram. Nodule segmentation: in the image of the set window level and window width bar, the anchor points of the segmentation line is set continuously along the junction of nodule and lung. The anchor size is the default of the software, and there is no interval between the anchor points.

Doctors manually segment Sn and determine VOI according to the following methods and standards: (1) specify that the window level of CT image is − 550HU and the window width is 1500HU to distinguish the boundary between SN and lung; (2) Anchor points are set continuously along the boundary between SN and lung, and the connecting line between anchor points is the dividing line; (3) VOI does not include continuous vessels and bronchus. If there are bronchus or vessels in the nodule, anchor points should be set along the edge of bronchus and vessels; (5) VOI includes residual truncated bronchi or small vacuoles scattered in SN (Figs. 1, 2).

A total of 490 Radiomics features were extracted from VOI, including 330 Gray Level Cooccurrence Matrix (GLCM), 33 Gray Level Run Length Matrix (GLRM), 55 Intensity Direct (ID), 49 Intensity Histogram^19^, 5 Neighbor Intensity Difference (NID), 18 Shape features^19–23^. The data supplement introduces the method and modification standard of VOI hook drawing, the method of Radiomics feature extraction and the detailed information of each parameter, supplement data for details.

Feature selection and machine learning classification model. We use the least absolute shrinkage selection operator (LASSO) regression to filter out invalid features from texture features, and the selected texture features are used to construct a classification model.

**Figure 3:**
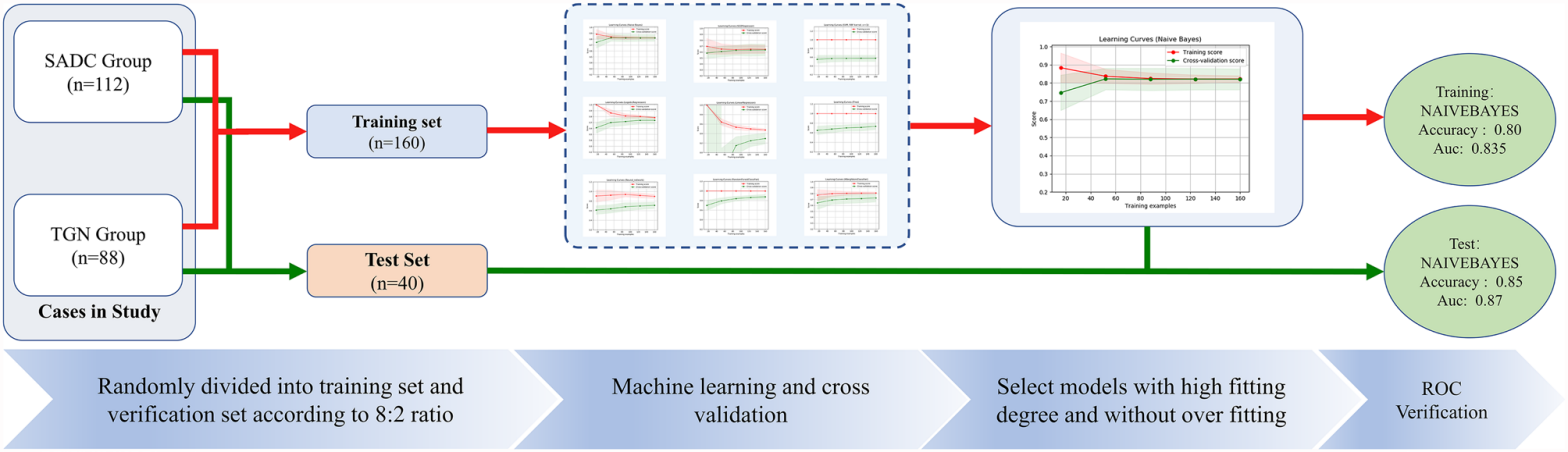
Machine learning flow chart. The samples after LASSO regression were randomly divided into a training group and test group at a ratio of 8:2. In the process of machine learning, 12 types of classifiers are used for training, and five times of cross-validation are performed during the learning process. Finally, the learning curve is drawn and the training score and verification score of each classifier is obtained; according to the learning curve of each classifier to score, select a classifier with a high degree of fit and no overfitting, and build and test a machine learning classification model.

This study uses Numpy and Sklearn packages to build a classification model on Python v3.8.6 platform. Firstly, multiple classifiers (exhausting all classifiers in Sklearn package) dynamic machine learning is carried out, and then the classifier with the highest classification accuracy and no over fitting is selected from the learning results to construct the classification model. Finally, the effectiveness of the classification model in differentiating invasive adenocarcinoma from tuberculous granuloma was evaluated. Results confusion matrix (classification table) was generated and expressed by area under curve (AUC) of receiver operating characteristic curve (Figs. 1, 3).

Models for CT sign classification and conventional imaging. Based on the CT signs of solitary pulmonary nodules, including the measured values of the most horizontal diameter (anteroposterior diameter, left–right diameter, upper and lower diameter), the average CT values of the central region, the vacuoles in the nodules, the outline of the nodules (lobulation), the spiculation of the nodules, the junction between the nodules and the lung tissue, the relationship between the nodules and the vessels, the relationship between the nodules and the bronchus, the relationship between the nodules and the adjacent pleura, the hilar or mediastinal lymph nodes, the hilar or mediastinal lymph nodes Multivariate logistic regression analysis was used to establish the classification model of CT parameters and CT signs^13^. The clinical CT diagnostic classification model was established by multiple logistic regression analysis, which combined the CT features and demographic data of patients. Two confusion matrices (classification tables) were generated, which were expressed by area under the curve (AUC) of the receiver operating characteristic curve and were used to predict the efficiency of distinguishing pulmonary invasive adenocarcinoma from tuberculous granuloma.

### Statistical analysis

The data of demographic and CT parameters and CT signs were analyzed by IBM SPSS v26.0 software (IBM, Redmond, WA, USA). The Chi-square test was used for counting data. An independent sample t-test was used for the measurement data meeting the normal distribution, otherwise, the Mann Whitney H-Test was used. The data of demographic and CT parameters and CT signs were further analyzed by binary logistic regression and the diagnostic model was established. The R language package used in this study is reported in the supplement. P < 0.05, with statistical significance.

## Results

**Figure 4:**
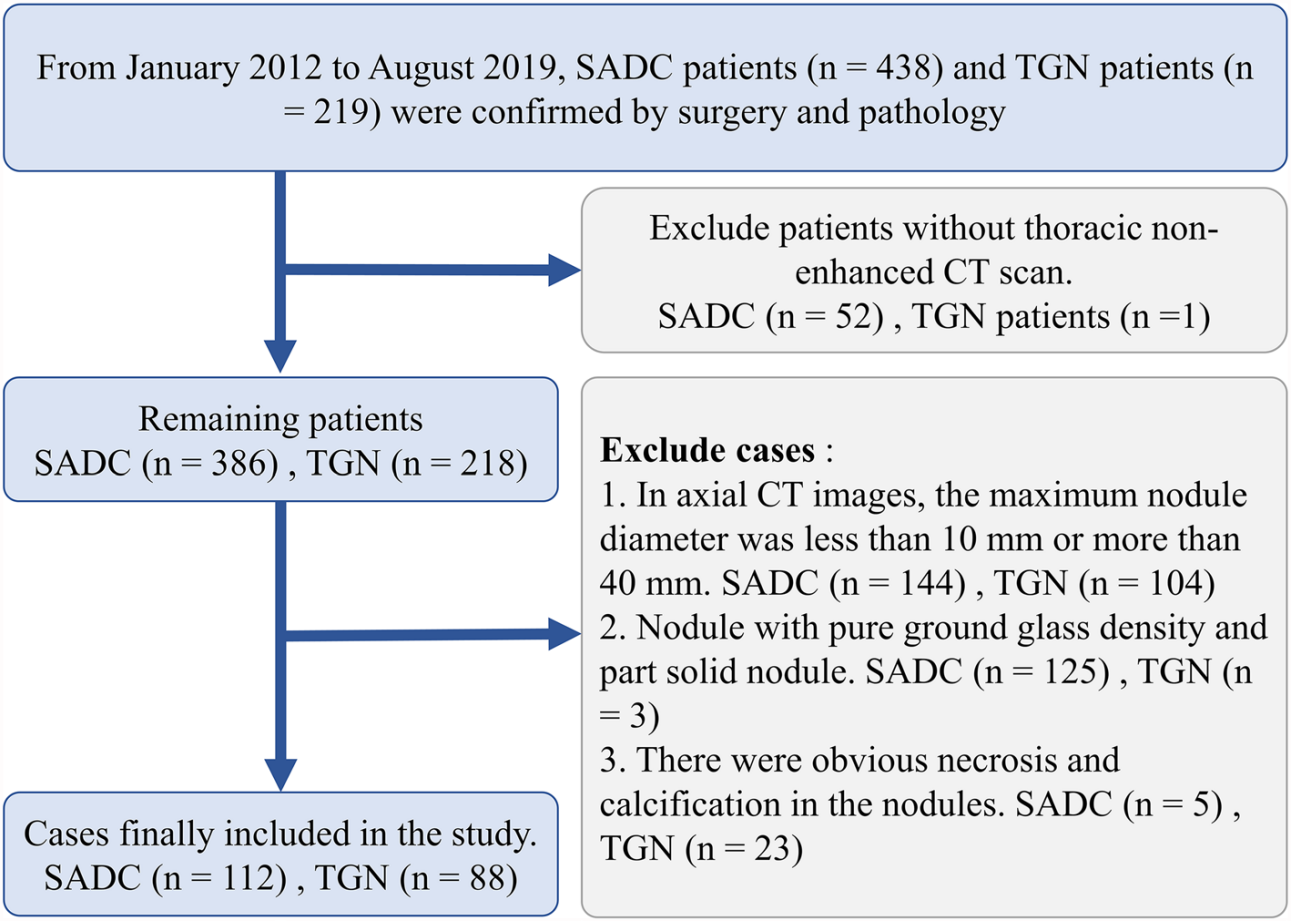
Flow chart of selecting case samples. Case elimination flowchart. 200 patients were selected according to the inclusion criteria.

**Figure 5:**
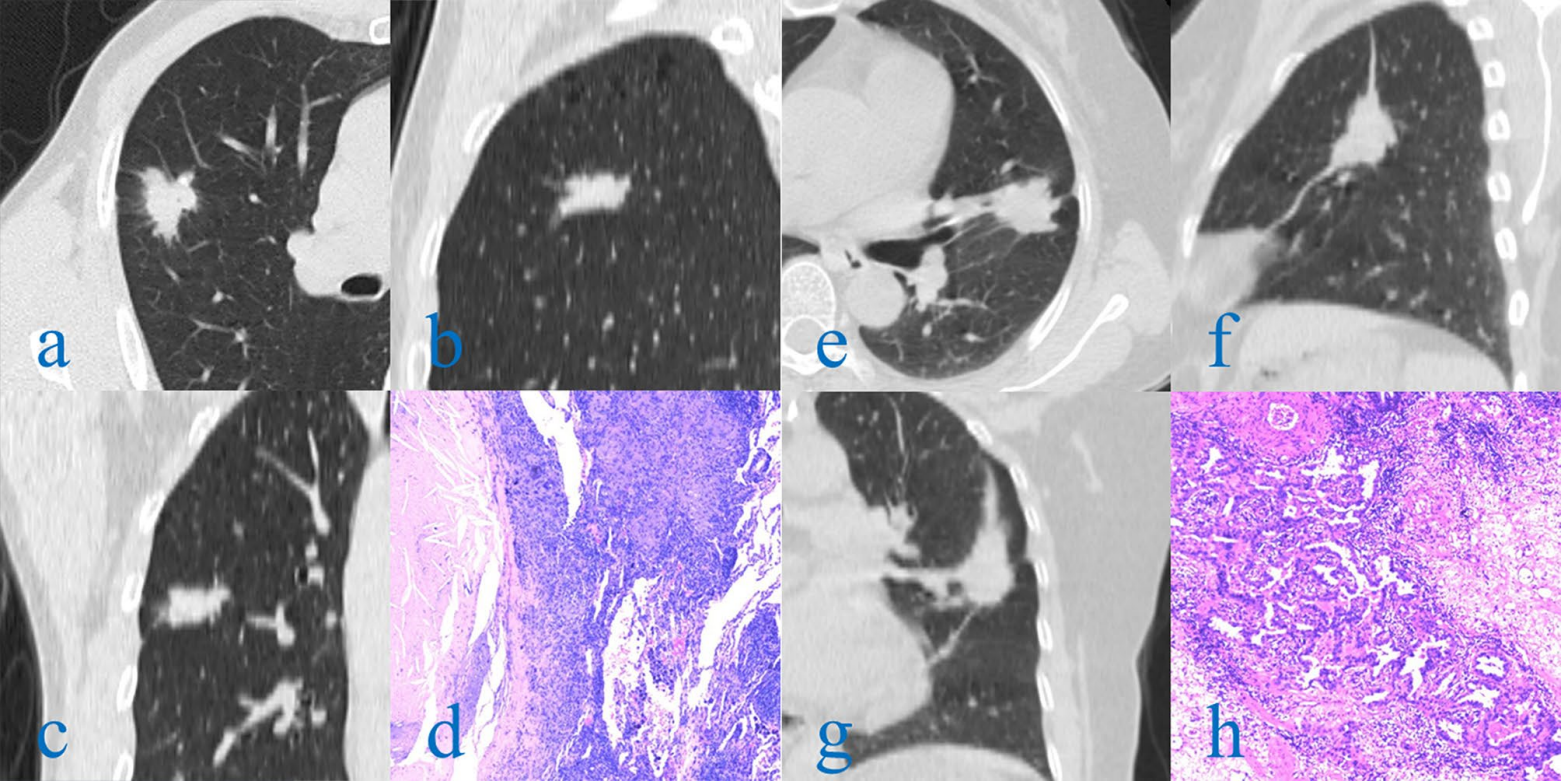
(**a**–**h**) Axial, Sagittal and Coronal CT images and pathological sections (HE, 400X, light microscope). A 65-year-old man underwent chest CT during "physical examination" and found right lung space-occupying lesions. The pathological diagnosis was tuberculous granulomatous nodule after operation. CT images showed superficial loculated nodules with rough edges, short and hard "burr", thin line like adhesion with pleura, and small blood vessels passing through the nodules (**a**–**c**). Pathological sections showed that the alveolar structure had been completely lost, and the multinucleated giant cells and epithelioid cells were disorderly accumulated (**d**). A 43-year-old female patient with coronary heart disease was diagnosed with left lung space-occupying lesion during coronary CTA. The pathological diagnosis was invasive adenocarcinoma after operation. CT images showed loculated, irregular nodules with smooth edge, long and soft "burr", pleural adhesion, vascular and bronchial convergence (**e**–**g**). Pathological sections showed that tumor cells with large nuclei and few cytoplasm accumulated along the alveolar wall (**h**).

Patients. In 657 patients with SADC and TGN, most of them were excluded according to the inclusion and exclusion criteria. Finally, 112 SADC patients and 88 TGN patients were still included in the study (Figs. 4, 5).

In the study of 200 patients, we conducted a simple statistical analysis of demographic data and unenhanced CT parameters and CT signs. The results are shown in Table 1. It showed that there were significant differences between SADC and TGN in Gender, Age, Smoking status, Smoking index, spiculated of nodule, and blood vessel in nodule (Tables 1).

**Table 1:** Biological features and CT features of all patients.

Variable	Sample	TGN	SADC	Statistics	P-value
Demographic					
Age		51.65 ± 11.77	58.52 ± 8.78	− 4.727	< 0.001
Gender				3.128	0.077
Female	105	40 (45.45%)	65 (58.04%)		
Male	95	48 (54.55%)	47 (41.96%)		
Smoking-status				53.46	< 0.001
Never	106	26 (29.55%)	80 (71.43%)		
Former	66	34 (38.64%)	32 (28.57%)		
Current	28	28 (31.82%)	0 (0.00%)		
Smoking-index (pick-year)				77.073	< 0.001
< 20	48	27 (30.68%)	54 (48.21%)		
20–50	21	21 (23.86%)	0 (0.00%)		
> 50	25	25 (28.41%)	0 (0.00%)		
CT features					
Cavity				2.06	0.151
No	154	72 (81.82%)	82 (73.21%)		
Yes	46	16 (18.18%)	30 (26.79%)		
Lobulated				0.355	0.551
None	37	18 (20.45%)	19 (16.96%)		
Slight	87	38 (43.18%)	49 (43.75%)		
Deep	76	32 (36.36%)	44 (39.29%)		
Edge				0.023	0.88
Clear	58	26 (29.55%)	32 (28.57%)		
Dim	142	62 (70.45%)	80 (71.43%)		
Around				0.702	0.402
Smooth	144	66 (75.00%)	78 (69.64%)		
Ragged	56	22 (25.00%)	34 (30.36%)		
Air bronchogram				0.002	0.961
No	139	61 (69.32%)	78 (69.64%)		
Yes	61	27 (30.68%)	34 (30.36%)		
Vessel			4.964	0.026	
No	3	3 (3.41%)	0 (0.00%)		
Yes	197	85 (96.5%)	112 (100%)		
Spiculation				6.823	0.009
None	72	40 (45.45%)	32 (28.57%)		
Fine	65	27 (30.68%)	38 (33.93%)		
Coarse	63	21 (23.86%)	42 (37.50%)		
Pleural depression				11.46	0.001
No	102	33 (37.50%)	69 (61.61%)		
Yes	98	55 (62.50%)	43 (38.39%)		
Lymph nodes				3.239	0.072
No	180	83 (94.32%)	97 (86.61%)		
Yes	20	5 (5.68%)	15 (13.39%)		

In this study, TGN patients were older than SADC patients (χ^2^ = 112.636, P < 0.01). There were 94 smokers (47%) and 106 never-smokers (53%). The difference in smoking rate between TGN patients and SADC patients was statistically significant (χ^2^ = 97.102, P < 0.01). Logistic regression model adjusted for age, nodular cavity, pleura depression and burr sign showed that the incidence rate of SADC increased among non-smokers relative to smokers (OR = 0.283, 95% CI: 0.147–0.545), while smoking was not associated with SADC incidence. The results of multivariate analysis are shown in Table 2.

**Table 2:** Logistic regression analysis of clinical CT.

	OR (95%CI)	P-value
Age	1.060 (1.018–1.103	0.005
Smoking-status	0.283 (0.147–0.545)	0.000
Smoking-index		
< 20	8,882,439	0.998
20–50	1.345	0.997
> 50	1.538	1.000
Cavity	4.234 (1.324–13.598)	0.015
Pleural depression	0.205 (0.078–0.539)	0.001
Spiculate	1.796 (1.080–2.988	0.024

**Figure 6:**
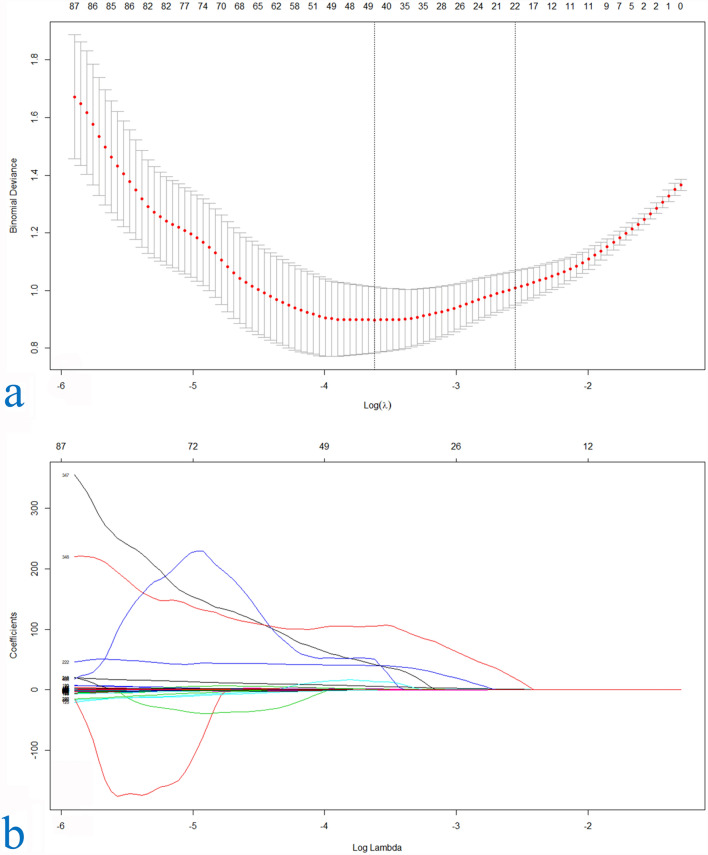
LASSO regression dimensionality reduction. Texture feature selection using the least absolute shrinkage and selection operator (LASSO) binary logistic regression model. (**a**) Tuning parameter (l) selection in the LASSO model used ten-fold cross-validation via minimum criteria. The area under the receiver operating characteristic (AUC) curve was plotted versus log(l). Dotted vertical lines were drawn at the optimal values by using the minimum criteria and the 1 standard error of the minimum criteria (the 1-SE criteria). An l value of 0.009, with log (l), 24.709 was chosen (1-SE criteria) according to ten-fold cross-validation. (**b**) LASSO coefficient profiles of the 490 texture features. A coefficient profile plot was produced against the log (l) sequence. A vertical line was drawn at the value selected using ten-fold cross-validation, where optimal l resulted in 23 nonzero coefficients.

Feature extraction and Radiomics signature building. Of the Radiomics characteristics, 23 potential predictors were screened out from 490 features based on 200 patients, with non-zero coefficients in the LASSO regression model (Fig. 6).

**Figure 7:**
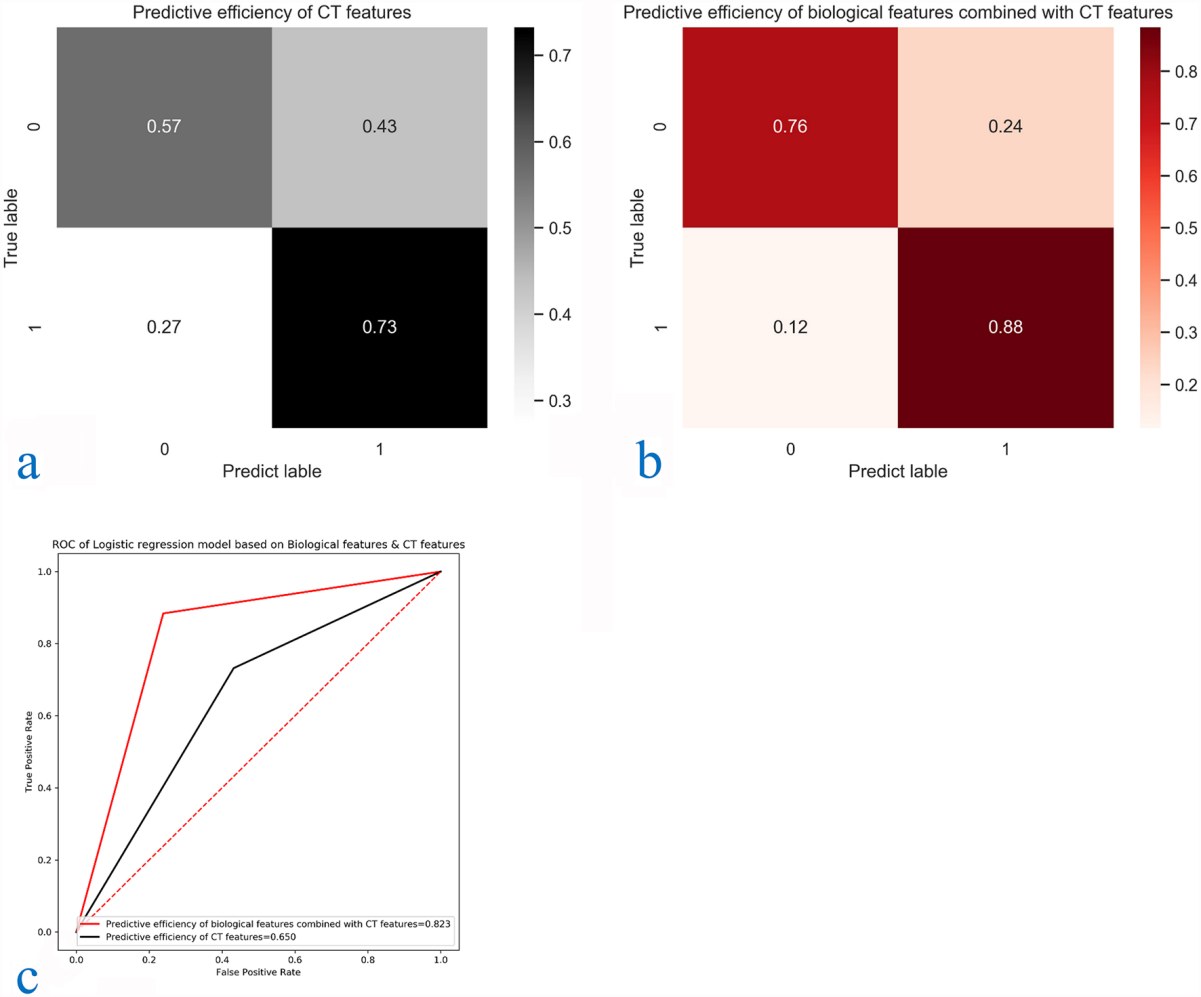
(**a**–**c**) Confusion matrix and ROC curve of CT features classification results and biological characteristics combined with CT features classification results.

Machine learning results and verification. The results of 23 selected texture features and 9 classifier learning curves are shown in the Supplement data, in which Stochastic gradient descend (SGD) regressor, Support vector machine (SVM), Logistic regression, Linear regression, Tree, Neural network, KNeighborsClassifier is insufficient fitting, and Random Forest classifier is overfitting. The learning process of Naïve Bayes shows that with the increase of samples, the fitting degree gradually tends to be unified, reaching the ideal classification results of the classifier. Some classifiers in Sklearn library have no fitting trend in the learning curve, so we will not repeat them here. Finally, the Naive Bayes classifier with AUC greater than 0.82 and training accuracy less than 1.000 in the cross-validation of the learning curve, and the training set and validation set tend to coincide at the end of the learning curve, is selected to construct the classification model of this study. We then evaluated the confusion matrix-related classification metrics of Naive Bayes. The accuracy of classification was 0.85. Sensitivity, specificity, PPV, and NPV were 0.736, 0.952, 0.933, and 0.80 respectively. The cross-validated AUC scores, AUC curve on the test datasets, and confusion matrix with normalization were shown in Fig. 7a–c and Table 3.

**Table 3:** Confusion matrix results of machine learning classification, CT only classification and clinical CT classification.

	AAC (%)	TPR (%)	TNR (%)	PPV (%)	NPV (%)	AUC	P	
NaiveBayes-Classifier								
Training (n = 160)	130/160 (81.2)	68/96 (70.8)	62/64 (96.9)	68/70 (97.1)	62/90 (68.9)	0.83	0.000	
Test (n = 40)	35/40 (85.0)	14/19 (73.7)	20/21 (95.2)	14/15 (93.3)	20/25 (80.0)	0.87	0.000	
Predictive efficiency of CT features								
(n = 200)	132/200 (66.0)	50/80 (62.5)	82/120 (68.3)	50/80(56.8)	82/112 (73.2)	0.65	0.000	
Predictive efficiency of biological features combined with CT features								
(n = 200)	166/200 (83.0)	67/80 (83.8)	99/120 (82.5)	21/34 (76.1)	99/112 (88.3)	0.82	0.000	

**Figure 8:**
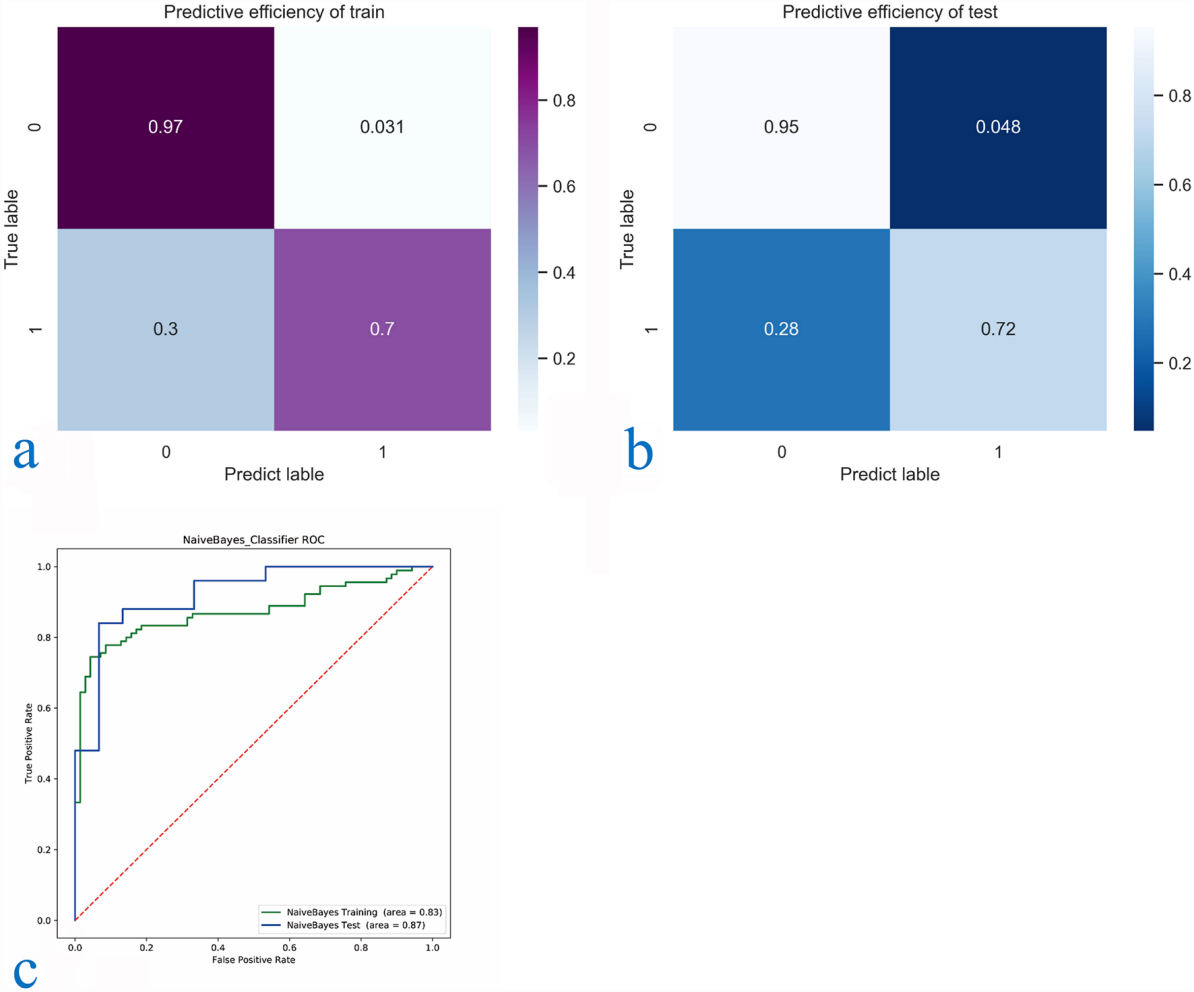
(**a**–**c**) Confusion matrix and ROC curve of classification results of the training group and test group of naive Bayesian classifier.

Apparent performance of the integration between the clinical markers and Radiomics in the training cohort. We also evaluated the confusion matrix classification measure based on CT parameters and CT signs alone and CT parameters and CT signs combined with biological information. The accuracy of the two classification models is 0.65/0.80. The sensitivity, specificity, PPV and NPV were 0.62/0.83, 0.68/0.82, 0.568/0.731 and 0.732/0.883, respectively. The AUC score of cross-validation, AUC curve on test data set and standardized confusion matrix are shown in Fig. 8a–c and Table 3.

Clinical application. The Confusion matrix analysis for the Radiomics signature and the clinical information is showed in Figs. 7, 8. The ROC curve showed that the Radiomics classification model had improved performance in the entire threshold range, compared with the single CT sign and CT sign combined with demographic classification model.

## Discussion

As far as we know, this is the first study to extract quantitative texture features from all sections non-enhanced CT images of SN in the differential diagnosis of SADC and TGN^15,24–26^. In this study, we designed a machine learning workflow based on Radiomics. Using 490 texture features extracted from the image, we established a machine learning classifier for the differential diagnosis of SADC and TGN and evaluated the performance of the classifier in the differential diagnosis.

In the past, the differential diagnosis of solid pulmonary nodules by discovering various features from the thoracic CT images containing a huge amount of information has brought great challenges to radiologists. Therefore, radiologists only focused on the typical features of nodule tissue images, rather than all the layers, including the whole lung parenchymal nodule images. One of the advantages of our method is that all CT images containing solid pulmonary nodules were included in the study. This allows a large amount of information contained in the entire CT image of solid pulmonary nodules that cannot be recognized by naked eyes to be used for differential diagnosis. This is a quantitative feature extracted from all levels of CT images of pulmonary solid nodules to predict the nature of pulmonary solid nodules. Therefore, it can provide more efficient and objective classification and prediction of solid nodules, and provide reliable basis for clinical decision-making.

An important part of our image processing technology is to select all layers of the nodule in the CT image so that all voxels of the nodule will be included in the study. Different from the normal lung tissue which is mainly composed of alveolar structure with relatively sparse cells, tumors and tuberculous granuloma with dense cell accumulation are shown as solid nodules in CT images, which are easy to be found by naked eyes. However, the CT images of solid nodules show limited features that can be recognized by naked eyes, resulting in the low efficiency of differential diagnosis. Texture features extracted from CT images can provide more biological information, which is expected to improve the efficiency of differential diagnosis. The solid nodule classifier we built utilizes 490 features extracted from CT images of solid nodules. These features include the local anatomical structure of the nodule (for example, the spatial size, surface area and CT value of the nodule) and more global patterns (for example, the texture composed of a planar pixel, the texture composed of three-dimensional spatial voxels)^16,27,28^. In order to improve the efficiency of machine learning in differential diagnosis, we use lasso regression as a benchmark of objective feature availability and select the most different features for machine learning. The least and effective texture features can help to improve the efficiency of trained radiologists in the differential diagnosis of solid nodules.

Different from the previous use of thoracic CT images with machine learning to differentiate pulmonary adenocarcinoma and tuberculous granuloma^29,30^, we realized automatic machine learning through the Python code. We design an automatic machine learning method to fully exploit the computing power of the computer, let the computer exhaust all possible machine learning classifier algorithms, use the learning curve to reproduce the dynamic process of classification learning of each classifier, and calculate the classification accuracy of each classifier, use the classification accuracy to evaluate the performance of each classifier, objectively select efficient classifiers, and construct a classification system model. Our model can effectively classify pulmonary solid nodules, and can assist trained radiologists in the differential diagnosis of pulmonary solid nodules.

The pathological basis of SADC and TGN is completely different: at present, it is considered that most SADC is derived from alveolar epithelium, and tumor cells are arranged in clusters or piles to grow into the cavity; the original alveolar structure is related to the development of adenocarcinoma, and the original alveolar wall structure is partially retained or extensively destroyed with remodeling, alveolar atrophy and collapse, and proliferation of fibrous tissue in the pulmonary septum, forming fibrous tissue of different sizes Small invasive lesions were common in the inner/peripheral area of fibrous scar area11. TGN is a kind of special chronic proliferative inflammation. It is a well-defined nodular lesion formed by the aggregation and proliferation of macrophages and their evolving cells (such as dermoid cells and multinucleated giant cells)^31^. The size of granulomas varies greatly, the fusion is more common, and the distribution is uneven. There are few reticular fibers in the central region. The evolution process of epithelioid cells around the nodules is different^32,33^. Because the epithelioid cells in tuberculous granuloma have biological manifestations like adenocarcinoma cells, such as disordered accumulation of cells in spatial arrangement and limited multi-directional differentiation, the CT parameters and CT signs of tuberculous granuloma in a certain growth stage are superficial lobulated solid nodules and pleural adhesion, which are like adenocarcinoma. In this study, we proved that SADC and TGN can be distinguished only according to the morphological features of CT images of solid nodules, including pleural adhesion, burr shape features, and mediastinal lymph nodes. However, the classification efficiency of these image features is low, which is basically invalid. Therefore, it is difficult to correctly recognize by naked eye observation of CT images. Because CT examination is routine in clinical practice, our classifier can effectively improve the accuracy of routine diagnosis.

We further improved the classification model of conventional CT diagnosis by combining CT image signs with demographic data. SADC and TGN can be effectively differentiated by age, smoking index, the small cavity of nodules, pleural adhesion, and burr. However, the classification deviates from people's common understanding, that is, lung adenocarcinoma with solid nodules is younger, the smoking index is small, and there are many small cavities Pleural adhesions are common and hard burr if radiologists based on common-sense diagnosis is likely to misjudge. Our machine learning classification model can effectively use a few texture parameters in the model, and can correctly classify solid nodules with high computational efficiency. This method is very suitable for analyzing numerous data and features. The accurate classification generated by our model can correct the clinical classification and enhance the accuracy of radiologists.

In this study, we found the main texture features of pulmonary adenocarcinoma and tuberculous granuloma with solid nodules. Glgm, Glrm, intensity direct and intensity histogram can distinguish the subtle differences of gray distribution and intensity between voxels in CT images due to the morphology and distribution of tumor cells or proliferative granuloma cells and differentiated tissues. Texture features quantify the correlation between two-dimensional and three-dimensional space of adjacent voxels in the region of interest. This indicates that the nodule's subtle difference is an important factor in determining the types of nodules. This partly reflects the local microanatomic structure (such as the distribution and arrangement of cells)^27^ and the overall pattern of tumor nuclei (such as the shape of nuclei and cytoplasm)^34^, which can improve the differential diagnosis efficiency.

One limitation of this study is that the developed model was not validated by an external validation team. This is because no matter the CT images of cases from other institutions or the CT images from Lung Image Database Consortium (LIDC) and Image Database Resource Initiative (IDRI) database are used, the CT scanning parameters and reconstruction parameters of these external cases are determined according to the daily practice of each institution, which may lead to the disunity of CT image specifications and the deviation of CT images^35^. For example, some radiologists are used to viewing images with low radiation dose, low window level, wide window width and a large amount of information, while some radiologists are used to viewing images with high radiation dose, high window level, narrow window width and a small amount of information. Although images with uniform scanning parameters and reconstruction mode are helpful to generate machine learning models, the performance of these diagnostic models in the actual clinical environment still needs to be explored. By continuously incorporating external data, verifying, and modifying the models, we can relax the scanning parameters and remodeling to expand the compatibility of the models, to achieve real promotion.

In conclusion, we have proved that the quantitative feature classifier based on CT plain scan images can successfully predict lung adenocarcinoma and tuberculous granuloma presenting as solid pulmonary nodules. This ability is superior to the current practice used by radiologists to differentiate solid pulmonary nodules by CT features alone. The objective characteristics related to benign and malignant also provide enlightenment for radiology research. Similar methods can also be applied to the radiology of other organs. Our method can promote the differential diagnosis based on conventional CT images, and thus contribute to the accurate treatment of pulmonary solid nodules and improve the prognosis and quality of life. However, this research has limitations such as the lack of a multi-center validation and the lack of a study of the effectiveness of enhanced CT Radiomics.

## Supplementary Information

Supplementary Information.

## Data Availability

The python code used in this study has been uploaded to GitHub. Inclusion and exclusion criteria and Radiomics feature extraction methodology are available in Supplementary Data.
