# Supplementary material for: A study on the differential of solid lung adenocarcinoma and tuberculous granuloma nodules in CT images by Radiomics machine learning
Source: Sci Rep. 2023 Apr 11;13:5853. doi: 10.1038/s41598-023-32979-6 (PMC10090156; doi:10.1038/s41598-023-32979-6)
Supplement: Supplementary file 1 — Supplementary Information. [file 41598_2023_32979_MOESM1_ESM.docx]

# “Radiomics Machine Learning for Differentiating solid lung adenocarcinoma and tuberculous granuloma nodules

**Appendix A1:** **Inclusion and exclusion criteria**

Inclusion criteria for patients with paraquat poisoning:

SADC patients and TGN patients were confirmed by surgery and pathology

Exclusion criteria:

1. The patient without chest unenhanced CT scan.;
2. In axial CT images, the nodule maximum diameter was less than 10 mm or more than 40 mm.；
3. Nodule with pure ground glass density;
4. There were obvious necrosis and calcification in the nodules;

**Appendix A2: ROI drawing methods and criteria.**

The standard of manual drawing is as follows: 1) the boundary between nodule and lung: the default CT image window is - 550hu, the window width is 1500hu, and the boundary between nodule and lung. 2) Draw anchors as continuous anchors. 3) VOI does not include blood vessels and bronchus. If there are bronchus or blood vessels in the nodule, avoid bronchus and blood vessels. 5) Small lungs scattered in bronchi or small vacuoles were included in VOI.

**Appendix A3: The packages of R software and Python used for statistical analysis**

Feature selection with R software (version 4.0.3; http://www.Rproject.org). The packages in R that were used in this study were as follows: Lasso binary logistic regression was done using the “glmnet” and "pROC" package.

Python software (version 3.7.4; https://www.python.org/ ）Write running code The python libraries used in this study are as follows: machine learning using "sklearn", "Pandas" and "numpy" libraries; confusion matrix analysis using "Pandas", "numpy" and "Seaborn" libraries; ROC (receiver operating characteristic) curve and confusion matrix drawing using "Matplotlib" library.

**Appendix A4: Radiomics feature extraction methodology**

In our study, a total of 490 imaging features for each patient were extracted. All radiomics characteristics were calculated automatically by non-commercial imaging biomarker explorer (IBEX) ，developed at MD Anderson Cancer Center (Houston, TX, USA) . All 490 features are divided into two orders and six categories: first order: Intensity-Direct histogram and Shape factor features; Second order texture features: gray level co-occurrence matrix (GLCM), gray level run length matrix (GLRL), Neighbor-Intensity-Difference (NID), Intensity-Direct. We preprocess image with average smooth of 3 pixels.

**The distribution of texture features is as follows:**

| **Category** | **Feature** | **Parameters** |
| --- | --- | --- |
| **Group-1（Feature 1-330）** | | |
| **Gray-Level-Cooccurence-Matrix 3** | | |
| Direction=0,45,90,135;  AdaptLimitLevel=0; GrayLimits=0,2100;  NumLevels=100;  Offset=1,4,7;  Symmetric=0; | AutoCorrelation |  |
|  | ClusterProminence |  |
|  | ClusterShade |  |
|  | ClusterTendendcy |  |
|  | Contrast |  |
|  | Correlation |  |
|  | DifferenceEntropy |  |
|  | Dissimilarity |  |
|  | Energy |  |
|  | Entropy |  |
|  | Homogeneity |  |
|  | Homogeneity2 |  |
|  | InformationMeasureCorr1 |  |
|  | InformationMeasureCorr2 |  |
|  | InverseDiffMomentNorm |  |
|  | InverseDiffNorm |  |
|  | InverseVariance |  |
|  | MaxProbability |  |
|  | SumAverage |  |
|  | SumEntropy |  |
|  | SumVariance |  |
|  | Variance |  |
| **Group-2（Feature 331-363）** | | |
| **Gray-Level-Run-Length-Matrix 3** | | |
| Direction=0,90;  GrayLimits=0,2100;  NumLevels=100; | GrayLevelNonuniformity |  |
|  | HighGrayLevelRunEmpha |  |
|  | LongRunEmphasis |  |
|  | LongRunHighGrayLevelEmpha |  |
|  | LongRunLowGrayLevelEmpha |  |
|  | LowGrayLevelRunEmpha |  |
|  | RunLengthNonuniformity |  |
|  | RunPercentage |  |
|  | ShortRunEmphasis |  |
|  | ShortRunHighGrayLevelEmpha |  |
|  | ShortRunLowGrayLevelEmpha |  |
| **Group-3（Feature 364-418）** | | |
| **Intensity-Direct** | | |
| ThresholdLow=0;  ThresholdHigh=8000;  ErosionDist=0; OnlyUseMaxSlice=0; | Energy |  |
|  | GlobalEntropy | NBins=256; RangeMin=0; RangeMax=4096; RangeFix=1; |
|  | GlobalMax |  |
|  | GlobalMean |  |
|  | GlobalMedian |  |
|  | GlobalMin |  |
|  | GlobalStd |  |
|  | GlobalUniformity | NBins=256; RangeMin=0; RangeMax=4096; RangeFix=1; |
|  | InterQuartileRange |  |
|  | Kurtosis |  |
|  | LocalEntropyMax | NHood=9; RangeMin=0; RangeMax=4096; |
|  | LocalEntropyMean | NHood=9; RangeMin=0; RangeMax=4096; |
|  | LocalEntropyMedian | NHood=9; RangeMin=0; RangeMax=4096; |
|  | LocalEntropyMin | NHood=9; RangeMin=0; RangeMax=4096; |
|  | LocalEntropyStd | NHood=9; RangeMin=0; RangeMax=4096; |
|  | LocalRangeMax | NHood=5; |
|  | LocalRangeMean | NHood=5; |
|  | LocalRangeMedian | NHood=5; |
|  | LocalRangeMin | NHood=5; |
|  | LocalRangeStd | NHood=5; |
|  | LocalStdMax | NHood=5; |
|  | LocalStdMean | NHood=5; |
|  | LocalStdMedian | NHood=5; |
|  | LocalStdMin | NHood=5; |
|  | LocalStdStd | NHood=5; |
|  | MeanAbsoluteDeviation |  |
|  | MedianAbsoluteDeviation |  |
|  | Percentile | Percentile=5,10,15,20,25,30,35,40,45,50,55,60,65,70,75,80,85,90, 95; |
|  | Quantile | Quantile=0.025, 0.25, 0.5, 0.75,0.975; |
|  | Range |  |
|  | RootMeanSquare |  |
|  | Skewness |  |
|  | Variance |  |
| **Group-4（Feature 419-467）** | | |
| **Intensity-Histogram** | | |
| NBins=256;  RangeMin=0;  RangeMax=4096;  RangeFix=1;  OnlyUseMaxSlice=0; | InterQuartileRange |  |
|  | Kurtosis |  |
|  | MeanAbsoluteDeviation |  |
|  | MedianAbsoluteDeviation |  |
|  | Percentile | Percentile=5,10,15,20,25,30,35,40,45,50,55,60,65,70,75,80,85,90, 95; |
|  | PercentileArea | Percentile=5,10,15,20,25,30,35,40,45,50,55,60,65,70,75,80,85,90, 95; |
|  | Quantile | Quantile=0.025, 0.25, 0.5, 0.75,0.975; |
|  | Range |  |
|  | Skewness |  |
| **Group-5（Feature 468-472）** | | |
| **Neighbor-Intensity-Difference 3** | | |
| NHood=3;  NHoodSym=1;  IncludeEdge=0;  AdaptLimitLevel=0; RangeMin=0; RangeMax=4096;  NBins=256; | Busyness |  |
|  | Coarseness |  |
|  | Complexity |  |
|  | Contrast |  |
|  | TextureStrength |  |
| **Group-6（Feature 473-490）** | | |
| **Shape** | | |
|  | Compactness1 |  |
|  | Compactness2 |  |
|  | Convex |  |
|  | ConvexHullVolume |  |
|  | ConvexHullVolume3D |  |
|  | Mass |  |
|  | Max3DDiameter |  |
|  | MeanBreadth |  |
|  | NumberOfObjects |  |
|  | NumberOfVoxel | EdgeVoxelFraction=0.5; |
|  | Orientation |  |
|  | Roundness |  |
|  | SphericalDisproportion |  |
|  | Sphericity |  |
|  | SurfaceArea |  |
|  | SurfaceAreaDensity |  |
|  | Volume | EdgeVoxelFraction=0.5; |
|  | VoxelSize |  |

**Appendix A5: Radiomics feature dimension reduction**

We use lasso regression to reduce the dimension. It’s a linear model that estimates sparse coefficients. It is useful in some contexts due to its tendency to prefer solutions with fewer non-zero coefficients, effectively reducing the number of features upon which the given solution is dependent. For this reason, Lasso and its variants are fundamental to the field of compressed sensing.

Mathematically, it consists of a linear model and a regularization term. The objective function is minimization:

$$\min_{w}\frac{1}{2n_{\text{samples}}}||Xw-y||_{2}^{2}+\alpha||w||_{1}$$

The lasso estimate thus solves the minimization of the least-squares penalty with $\alpha||w||_{1}$ added, where is a constant and $||w||_{1}$ is the -$\mathcal{l}_{1}-$norm of the coefficient vector.

**Appendix A6: The best classification method**

Naive Bayes methods are a set of supervised learning algorithms based on applying Bayes’ theorem with the “naive” assumption of conditional independence between every pair of features given the value of the class variable. Bayes’ theorem states the following relationship, given class variable *y* and dependent feature vector x_1_ through x_n,_

Because all kinds of texture features are continuous variables, we use the Gaussian Naive Bayes (GaussianNB).

$$P(x_{i}\mid y)=\frac{1}{\sqrt{2\pi\sigma_{y}^{2}}}\exp(-\frac{(x_{i}-\mu_{y})^{2}}{2\sigma_{y}^{2}})$$

The parameters $\sigma_{y}$ and $\mu_{y}$ are estimated using maximum likelihood.

**Appendix Figure A1. Patient enrollment flow chart
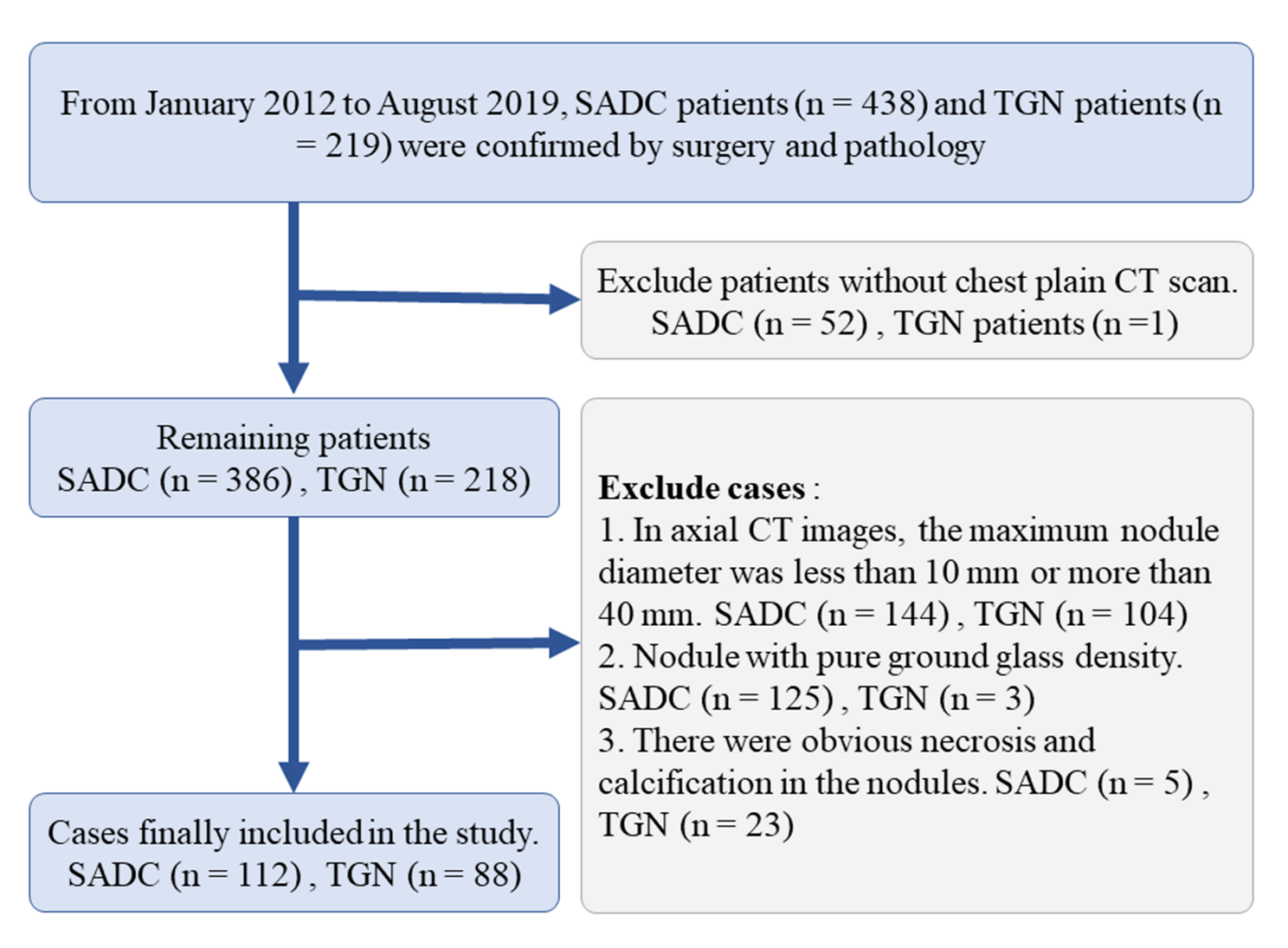
**

**
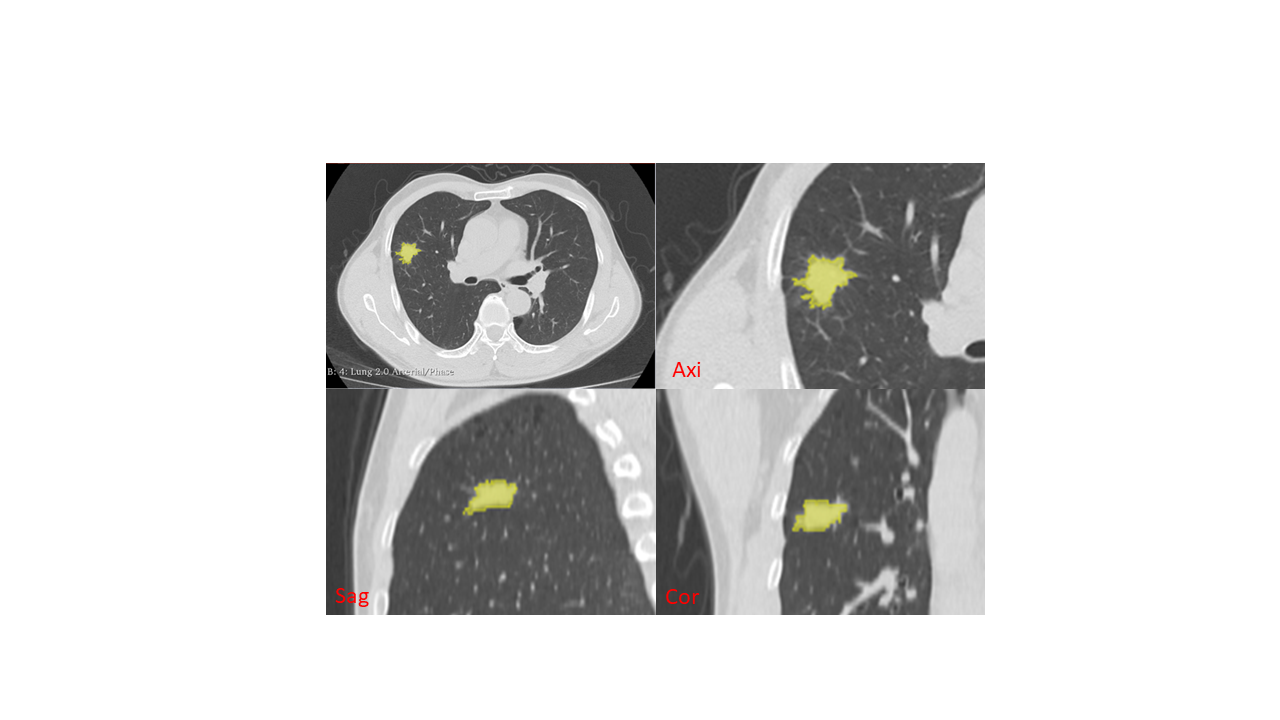

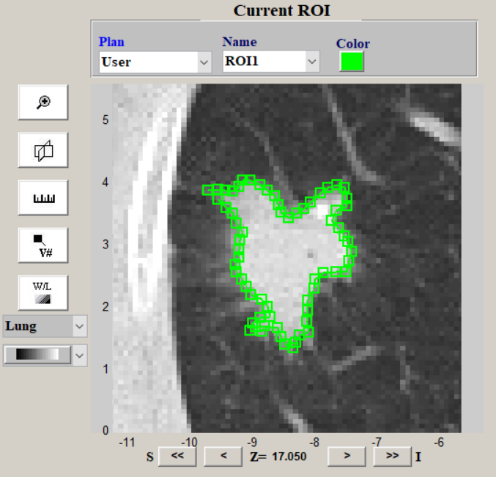
Appendix Figure A2.** Image segmentation diagram.

A：Nodule segmentation: in the image of the set window level and window width bar, the anchor points of segmentation line are set continuously along the junction of nodule and lung. The anchor size is the default of the software, and there is no interval between the anchor points.

**Appendix Figure A3.** Learning curves of various classifiers in machine learning


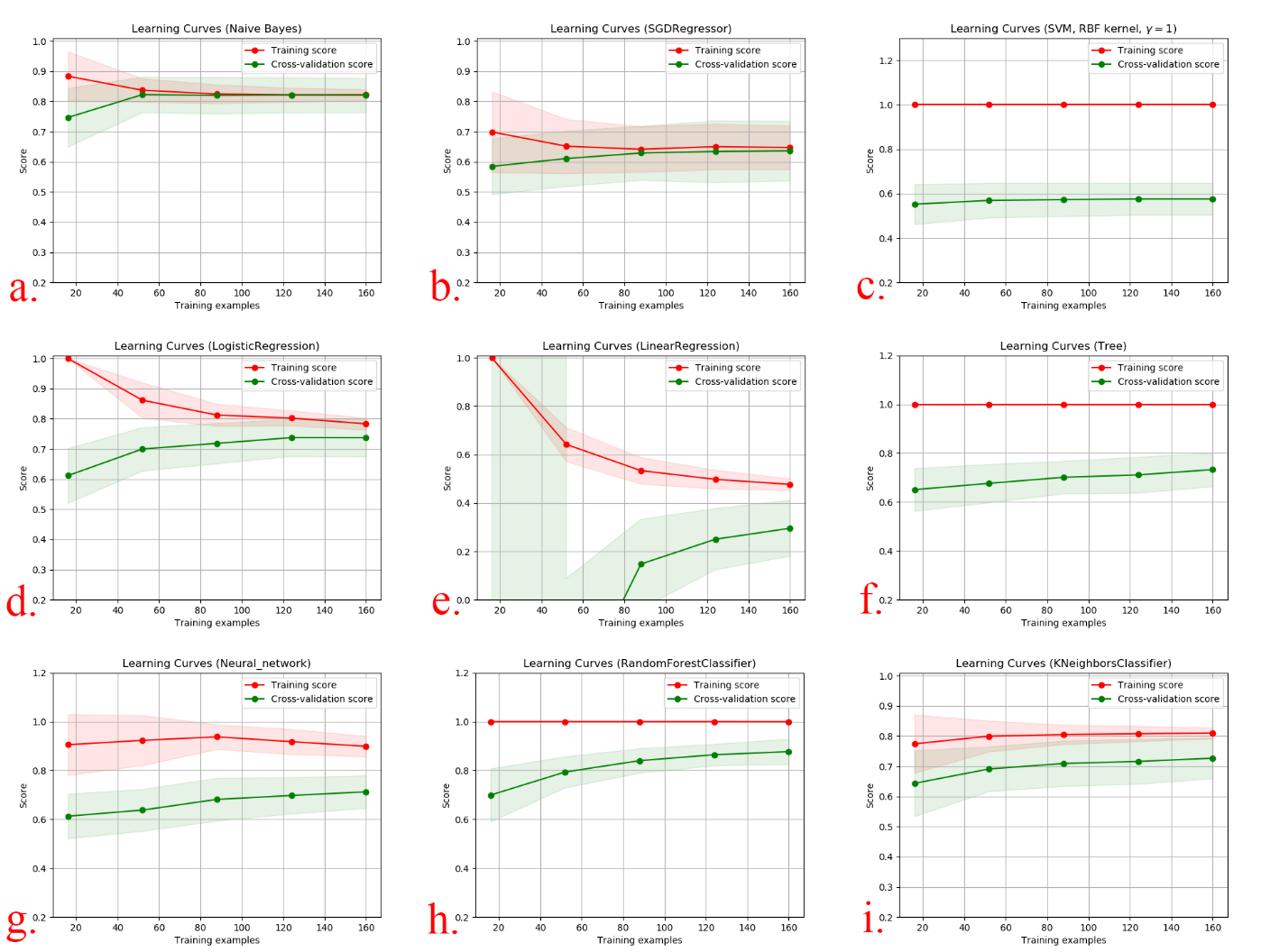


| **Group 1**  **GrayLevelCooccurenceMatrix3** | -333-1AutoCorrelation |
| --- | --- |
|  | -333-4Correlation |
|  | 90-4Correlation |
|  | 135-4Correlation |
|  | 90-7Entropy |
|  | 45-4InformationMeasureCorr2 |
|  | 45-4SumAverage |
|  | 90-4SumAverage |
|  | 90-7SumAverage |
|  | 45-7SumVariance |
|  | 135-4SumVariance |
| **Group 2**  **GrayLevelRunLengthMatrix3** | -333RunLengthNonuniformity |
|  | 90RunLengthNonuniformity |
| **Group 3**  **IntensityDirect** | LocalRangeMean |
|  | LocalRangeMin |
|  | LocalStdMean |
|  | LocalStdMedian |
|  | 15Percentile |
|  | 0.975Quantile |
| **Group 4**  **IntensityHistogram** | 10Percentile |
|  | 15Percentile |
|  | 10PercentileArea |
|  | 0.25Quantile |

**Appendix Table A5:** **Texture features involved in machine learning after dimension reduction.**

**Appendix Table A6:** **The accuracy score of each classifier in the process of machine learning.**

| Naive Bayes |  | |  | |  | |  | |  |
| --- | --- | --- | --- | --- | --- | --- | --- | --- | --- |
| Training_scores | 0.88375±0.08149962 | | 0.83711538±0.03860119 | | 0.82454545±0.03129934 | | 0.82145161±0.02315008 | | 0.822±0.01786407 |
| Cross-validation score | 0.74675±0.09718893 | | 0.8225±0.05900212 | | 0.82025±0.06069339 | | 0.8215±0.05884514 | | 0.82075±0.05734272 |
| SGDRegressor |  | |  | |  | |  | |  |
| Training_scores | 0.704375±0.14680317 | | 0.6575±0.08985915 | | 0.64318182±0.08975115 | | 0.65048387±0.07137183 | | 0.66675±0.06605443 |
| Cross-validation score | 0.578±0.09521554 | | 0.6125±0.09769212 | | 0.6085±0.094685 | | 0.63225±0.09587068 | | 0.6535±0.08351796 |
| SVM |  | |  | |  | |  | |  |
| Training_scores | 1±0 | | 1±0 | | 1±0 | | 1±0 | | 1±0 |
| Cross-validation score | 0.55225±0.09042227 | | 0.56925±0.07817089 | | 0.57275±0.07492455 | | 0.57575±0.07189011 | | 0.57575±0.07189011 |
| Kneighbors Classifier |  | |  | |  | |  | |  |
| Training_scores | 0.774375±0.09637776 | | 0.79980769±0.05117661 | | 0.80522727±0.03226232 | | 0.80798387±0.02528956 | | 0.81±0.01828592 |
| Cross-validation score | 0.6435±0.10923713 | | 0.691±0.07403715 | | 0.70925±0.07522092 | | 0.716±0.07445804 | | 0.72675±0.06729181 |
| Logistic Regression |  | |  | |  | |  | |  |
| Training_scores | 1±0 | | 0.86153846±0.05870899 | | 0.81238636±0.03659356 | | 0.80217742±0.02489455 | | 0.78325±0.01974209 |
| Cross-validation score | 0.6115±0.09085841 | | 0.69975±0.07224048 | | 0.7185±0.06667271 | | 0.7375±0.06159342 | | 0.73725±0.06229918 |
| LinearRegression |  | |  | |  | |  | |  |
| Training_scores | 1±0 | | 0.64189072±0.07027616 | | 0.53378765±0.0549181 | | 0.49768404±0.03875221 | | 0.47695255±0.02437727 |
| Cross-validation score | (341.15) | | (0.48) | | 0.147770634±0.185155069 | | 0.250352065±0.127122918 | | 0.295569118±0.115785264 |
| Tree |  | |  | |  | |  | |  |
| Training_scores | 1±0 | | 1±0 | | 1±0 | | 1±0 | | 1±0 |
| Cross-validation score | 0.65075±0.08473894 | | 0.67525±0.07529069 | | 0.6945±0.07677728 | | 0.71325±0.06914252 | | 0.72±0.07185054 |
| Neural_network |  | |  | |  | |  | |  |
| Training_scores | 0.896875±0.13789007 | | 0.93211538±0.09728013 | | 0.92409091±0.0641795 | | 0.92040323±0.04309282 | | 0.89725±0.05286569 |
| Cross-validation score | 0.58625±0.09652558 | | 0.65525±0.07333954 | | 0.6695±0.0862105 | | 0.6965±0.08624239 | | 0.72025±0.06952473 |
| RandomForestclassifier | |  | |  | |  | |  | |
| Training_scores | 1±0 | | 1±0 | | 1±0 | | 1±0 | | 0.9996875±0.00136216 |
| Cross-validation score | 0.69925±0.10876896 | | 0.79375±0.06397021 | | 0.84025±0.05011175 | | 0.8645±0.04418427 | | 0.87725±0.05197295 |
